# The influence of social exclusion on prosocial behavior of college students: the role of relational need threat and regulatory focus

**DOI:** 10.3389/fpsyg.2024.1384279

**Published:** 2024-04-24

**Authors:** Chunna Hou, Shuyu Li, Haoling Shi, Zhijun Liu

**Affiliations:** ^1^School of Psychology, Northeast Normal University, Changchun, China; ^2^School-Based Mental Health Center, The High School Affiliated to Shandong University, Jinan, China; ^3^Sociology Department, Changchun University of Science and Technology, Changchun, China

**Keywords:** social exclusion, prosocial behavior, relational need threat, regulatory focus, promotion orientation, prevention orientation

## Abstract

The present study investigated the impact of social exclusion on prosocial behavior, examining the roles of relational need threat and regulatory focus. Utilizing a questionnaire study with 483 participants (Study 1) and an experimental study with 100 participants (Study 2), we found that (1) social exclusion negatively predicted prosocial behavior; (2) relational need threat fully mediated the relationship between social exclusion and prosocial behavior; and (3) regulatory focus, categorized as either promotion or prevention, moderated this relationship in opposite directions. In conclusion, our findings reveal that social exclusion does indeed trigger prosocial behavior. Meanwhile, relational need threat and regulatory focus have a co-action impact on this process. These findings have been carefully discussed within the frameworks of the temporal need-threat model and the cognitive-affective personality system theory.

## 1 Introduction

Prosocial behavior encompasses positive social behaviors occurring in interpersonal interactions, such as helping, donating, sharing, and comforting (Eisenberg et al., 2015). Prosocial behavior not only is beneficial to others and society, but also has a positive impact on the mental health of both the participants and recipients, as well as on the development of human society (El-Khodary and Samara, 2019; Lott et al., 2020). For university students, engaging in prosocial behavior can significantly contribute to achieving academic honors, promoting life satisfaction, enhancing social adaptability, and even gaining a competitive edge (Bian and Wu, 2023).

The impact of external (environmental) and internal factors (emotional, cognitive, and trait) on prosocial behavior consistently remains a primary focus for scholars (Ding et al., 2018; Hughes et al., 2018). Among these factors, as an external contextual factor, the impact of social exclusion on prosocial behavior has always been inconsistent. Although most studies have shown that exclusion behavior reduces cooperation, some individuals still tend to be prosocial after being excluded (Derfler-Rozin et al., 2010; Walasek et al., 2019; Quarmley et al., 2022). Despite inconsistency in previous findings, understanding the motivational factors driving prosocial behavior following social exclusion is imperative.

The Cognitive-Affective Personality System (CAPS) proposes that prosocial behavior in individuals is influenced by the interplay of external contexts such as culture (Kärtner and Keller, 2012), cognitive processes and emotional responses (Carlo et al., 2010; de Hooge et al., 2011; Christner et al., 2022), as well as personality traits (see the meta-analysis of Thielmann et al., 2020). These findings provide insights into the intervention strategies for promoting prosocial behavior. Prior literature has demonstrated the influence of cognition and emotion on prosocial behavior (Rahal and Fiedler, 2022). Recent research has uncovered that music therapy might enhance prosocial behavior by affecting these two factors, especially among individuals who have undergone social exclusion (Martí-Vilar et al., 2023). Within the CAPS framework, the current study aims to integrate contextual, cognitive, and personality factors to explore their impact on prosocial behavior, particularly following experiences of social exclusion.

Given the link between relational need threat and prosocial behavior as suggested by the temporal need-threat model, this study incorporates self-regulatory orientation factors to understand how individuals adjust their prosocial behavior in the face of external exclusion situations.

### 1.1 Social exclusion and prosocial behavior

Previous literature suggests that prosocial behavior can be affected by social exclusion (Tu et al., 2022), leading to challenges in fulfilling personal belonging needs and social requirements (Williams, 2007). It is worth noting that there is still controversy among scholars regarding the influence of social exclusion. Several studies indicate that social exclusion inhibits individuals’ prosocial behavior. Specifically, individuals who have experienced social exclusion are observed to engage in less prosocial behavior in helping tasks and maintain greater social distance from their peers (e.g., sitting further apart) (Kothgassner et al., 2017). Additionally, they demonstrate reduced willingness to donate and contribute fewer actual donations. Scholars suggest that this might be attributed to social exclusion impeding an individual’s emotional capacity (Lee and Park, 2019). In other words, psychological frustration resulting from social exclusion may lead to emotional numbness as a self-protective mechanism to avoid further distress, ultimately inhibiting individuals’ ability to engage in prosocial actions (Twenge et al., 2007; Le et al., 2020; Shao et al., 2023).

Alternatively, some scholars argue that social exclusion can actually promote prosocial behavior among individuals. Previous research conducted with Spanish college students has shown that participants engaged in more donation behaviors following experiences of social exclusion (Cuadrado et al., 2016). Similarly, Chinese college students have demonstrated an increase in prosocial behaviors after encountering social exclusion (Fang and Chang, 2023). Scholars suggest that this may be linked to the notion that the experience of exclusion heightens an individual’s anticipation of forming social connections, thus leading to an increase in prosocial behavior (Carter-Sowell et al., 2008).

### 1.2 Relationships between social exclusion, relational need threat and prosocial behavior

The relational need threat may serve as one underlying cause of the previously mentioned divergence. Relational needs encompass basic human needs associated with belonging and self-esteem. According to the temporal need-threat model, these needs are centered on social relations and manifest as individuals’ desire for harmonious, intimate, and stable social connections. When these needs are threatened, they can impact an individual’s engagement in prosocial behavior (Williams, 2009).

Social exclusion implies damage to social relationships, prompting individuals to reconnect with reality to address their threatened relational needs. Prosocial behavior, by enhancing the likelihood of societal reintegration and gaining favor with others, may be the preferred method for individuals experiencing exclusion to bolster their relational needs (Ren et al., 2018). When relational needs are threatened, individuals tend to engage in prosocial behavior to address these needs by connecting with others (Wesselmann et al., 2015; Ren et al., 2018).

However, not all individuals facing threats to relational needs exhibit prosocial behavior. When individuals feel excluded, their relational needs are compromised, and the fundamental drive to form positive and amicable social bonds is disrupted. This disruption may lead to negative judgments about other group members and themselves, undermining the foundation for prosocial behavior (Twenge et al., 2007).

In conclusion, relational needs may play a critical role in shaping individuals’ behavioral responses following experiences of social exclusion (Chen et al., 2017). Therefore, we expect that the relational need threat may act as a mediating variable in determining how social exclusion impacts prosocial behavior.

### 1.3 The role of regulatory focus in the relationships among social exclusion, relational need threat, and prosocial behavior

Regulatory focus may also contribute to the variation in the effect of social exclusion on prosocial behavior. Regulatory focus pertains to the specific self-regulatory mode or tendency that individuals display while pursuing goals. It encompasses two orientations: promotion orientation and prevention orientation (Higgins, 1997; Higgins et al., 2001). Individuals with a promotional orientation possess a strong desire or aspiration for advancement, aiming to achieve higher status or accomplishment in their work, studies, or other aspects. They are sensitive to the presence or absence of positive outcomes, more inclined to empathize with group members, experience more positive emotions, and are more likely to exhibit prosocial behavior (Lee and An, 2023). Conversely, individuals with a prevention orientation are more concerned with security needs, view the desired end state as a duty and responsibility, and are sensitive to the possible presence or absence of negative outcomes. They are worry about rejection in interpersonal relationships and tend to adopt more indifferent strategies; hence, they are less likely to demonstrate prosocial behaviors (Gao et al., 2023). This perspective is supported by literature. For instance, participants with a promotion orientation opt to donate more frequently and in larger amounts than those with a prevention orientation (Gino and Margolis, 2011).

According to the CAPS theory, individual differences exhibited in a given situation depend on the influence of personality factors (Mischel and Shoda, 1995). Scholars propose that regulatory focus, as a personality factor, can play a moderating role in how social exclusion influences behavior (Adams and Tyler, 2021). Moreover, previous studies indicate that individuals with different regulatory focuses may display different behaviors when facing threats to their relational needs. For example, individuals with a promotion orientation tend to have higher self-esteem than those with a prevention orientation (Liu and Yao, 2019). Additionally, individuals with a promotion orientation feel a stronger sense of belonging in social networks and view this belonging as a key factor of happiness (Krishen et al., 2019).

This implies that the interaction between regulatory focus and relational need threat may lead to differences in prosocial behaviors, especially following social exclusion. Indeed, scholars have highlighted that the interaction between personality and cognitive factors impacts prosocial behaviors (Mischel and Shoda, 1995). However, it is noteworthy that relational need threat, as an immediate reaction subsequent to social exclusion, represents a widespread response during this stage and remains relatively unaffected by personality factors. Consequently, we hypothesize that regulatory focus plays a moderating role in the mediation model previously mentioned; it serves as a moderator between relational need threat and prosocial behavior. Specifically, under relational need threat, individuals with a promotion orientation are expected to exhibit more prosocial behaviors, while those with a prevention orientation are expected to demonstrate fewer prosocial behaviors.

Drawing from the temporal need-threat model, our study integrates the CAPS theory and aims to explore the underlying mechanisms through which relational need threat and regulatory focus exert their influence in the process of social exclusion affecting prosocial behavior. The two specific hypotheses, verified through questionnaires and laboratory examinations, are outlined as follows:

*Hypothesis 1*: Relational need threat plays a mediating role in the relationship between social exclusion and prosocial behavior.

*Hypothesis 2*: Regulatory focus moderates the relationship between relational need threat and prosocial behavior in the proposed mediation model.

**Figure 1:**
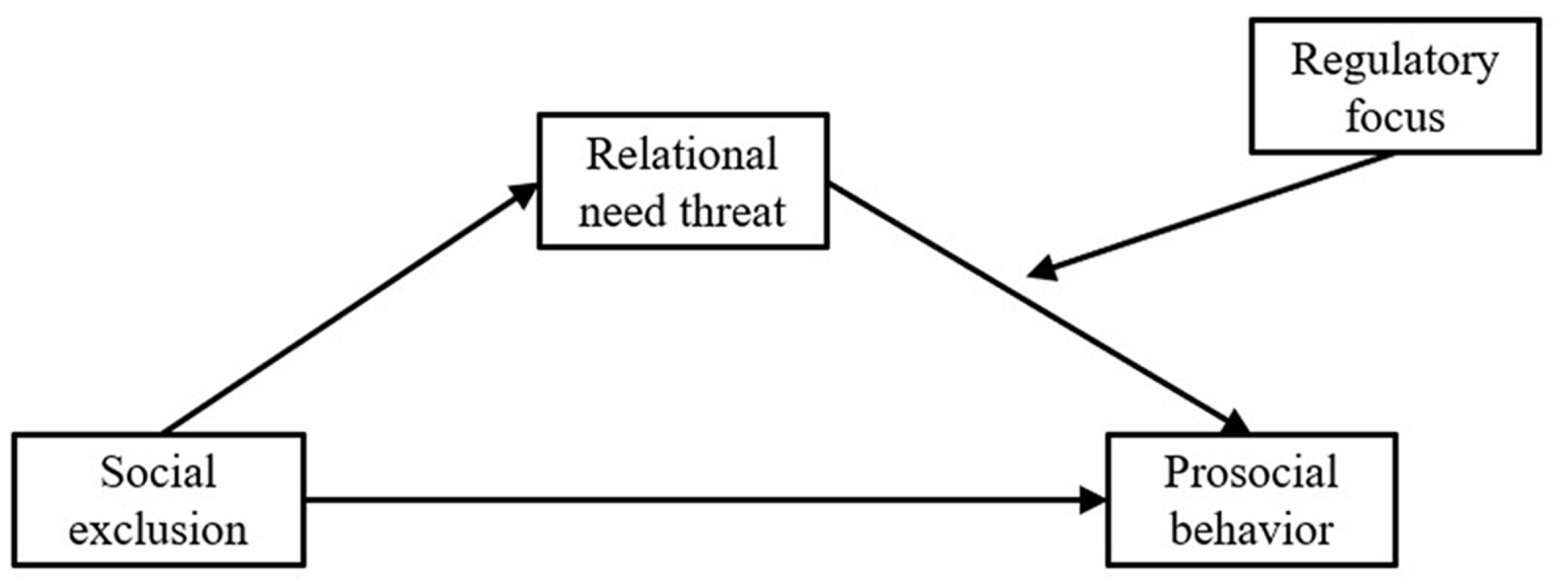
The moderated mediation effect model diagram.

The model diagram illustrating the relationships among variables is presented in Figure 1.

## 2 Study 1

According to regulatory focus theory, both promotion orientation and prevention orientation can be influenced by family environment, parenting style, and historical experience, leading to stable personality traits known as dispositional regulatory focus. In addition, both promotion orientation and prevention orientation can also be activated by environmental factors, resulting in a temporary psychological state that impacts individuals’ behavioral performance, referred to as situational regulatory focus (Higgins, 1997). In Study 1, we utilized questionnaire surveys to assess dispositional regulatory focus. The aim of this study was to examine how individuals with different orientations of regulatory focus respond to social exclusion, which threatens their relational needs, and how it influences prosocial behavior.

### 2.1 Method

#### 2.1.1 Participants

In this study, a total of 483 college students were recruited randomly from colleges and universities through an online platform. Due to missing or invalid responses, 63 participants were excluded from the analyses. Therefore, the final sample consisted of 420 participants (237 females, 56.4%). The mean age was 21.05 years (SD = 2.04). The study was approved by the Academic Ethics Committee of the College of Psychology of ****** University. The participants provided their written informed consent to participate in this study.

#### 2.1.2 Measures

##### 2.1.2.1 Social exclusion

Social exclusion was assessed using the Social Exclusion Scale, a 19-item self-report questionnaire (Wu et al., 2013). All the items were rated on a five-point Likert scale (from 1 = “not true” to 5 = “certainly true”). In this study, the total alpha coefficient was 0.97.

##### 2.1.2.2 Prosocial behavior

We employed the Prosocial Tendencies Measurement Scale, developed by Kou et al. (2007), to measure prosocial behaviors. A meta-analysis indicated that the reliabilities of the PTM were acceptable (Reig-Aleixandre et al., 2023). The scale comprised 26 items, each scored on a 5-point Likert scale ranging from 1 = “not true” to 5 = “certainly true.” The Cronbach’s alpha coefficient for internal consistency in this study was 0.95.

##### 2.1.2.3 Relational need threat

It was assessed using the Fundamental Need Threat Scale developed by Wirth et al. (2010). The scale consisted of 10 items, with responses scored on a 5-point Likert scale ranging from 1 = “not true” to 5 = “certainly true.” The Cronbach’s alpha coefficient for internal consistency in the current study was 0.93. Additionally, the variables of relational need threat were categorized into high and low groups using mean ± standard deviation.

##### 2.1.2.4 Regulatory focus

We used the Regulatory Focus Questionnaire, revised by Yao et al. (2008), to measure two dimensions of regulatory focus: promotion motivation (6 items) and prevention motivation (4 items). Participants rated each item on a five-point Likert scale, ranging from 1 = “not true” to 5 = “certainly true.” In this study, the reliability of the subscales was 0.81 and 0.84, respectively, and the total alpha coefficient was 0.81. Similarly, we utilized mean ± standard deviation to categorize the regulatory focus variable into high and low groups.

### 2.2 Data analysis

SPSS23.0 software and the SPSS PROCESS macro program were used for data processing. First, we computed descriptive statistics and conducted Pearson correlations. Second, after all the data were standardized, based on 5,000 bootstrap samples (Hayes and Scharkow, 2013), the mediating effect of relational need threat was analyzed using the PROCESS macro (Model 4) developed by Hayes (2013). Third, based on 5,000 bootstrap samples (Hayes and Scharkow, 2013), we used the PROCESS macro (Model 14) to examine whether regulatory focus moderated this mediation process. The effects are significant when the confidence intervals exclude zero.

### 2.3 Results

#### 2.3.1 The common method variance test

During the data collection, the participants were informed that their responses would remain anonymous, and some items were reverse-coded. After the data collection was complete, Harman’s one-factor test was employed to detect common method variance. The results of the exploratory factor analysis (EFA) revealed that the first factor explained 33.84% of the variance, which was less than the critical value of 40%. Consequently, there was no significant common method variance in this study.

#### 2.3.2 The mediating role of relational need threat between social exclusion and prosocial behavior

The PROCESS Model 4 of Hayes (2013) was employed to test the mediating role of relational need threat between social exclusion and prosocial behavior. Table 1 shows the descriptive statistics and correlations between variables.

**Table 1:** Means, standard deviations, and correlations among variables.

	*M*	*SD*	1	2	3	4	5
1. Social exclusion	42.21	16.60	–				
2. Prosocial behavior	95.42	16.47	−0.40^***^	–			
3. Relational need threat	25.72	9.12	0.64^***^	−0.61^***^	–		
4. Promotion	20.02	4.47	−0.36^***^	0.44^***^	−0.44^***^	–	
5. Prevention	14.51	3.60	−0.03	−0.02	0.12^*^	0.24^***^	–

^*^p < 0.05; ^***^p < 0.001.

First, social exclusion was found to have a significant negative predictive effect on prosocial behavior (*β* = −0.37, *t* = −4.90, *p* < 0.001). Second, the positive predictive effect of social exclusion on relational need threat was also significant (*β* = 0.62, *t* = 8.57, *p* < 0.001). Finally, the bootstrap indirect effect of social exclusion on prosocial behavior through relational need threat was −0.37, with 95% CI [−0.47, −0.29], which did not contain zero. This finding was consistent with the criteria defined in Preacher’s mediation model (see Table 2). It is noteworthy that the negative predictive effect of social exclusion on prosocial behavior became non-significant after incorporating relational need threat as a mediating variable in the equation (*β* = −0.01, *t* = −0.15, *p*>0.05). This suggests that relational need threat serves as a complete mediator in the relationship between social exclusion and prosocial behavior (see Table 2), thereby confirming Hypothesis 1.

**Table 2:** Testing the mediation effect of relational need threat on the relationship between social exclusion and prosocial behavior.

Regression equation		Fitting index		Coefficient significance	
Outcome variable	Predictor variable	*R^2^*	*F*	*β*	*t*
PSB	SE	0.17	27.87^***^	−0.37	−4.90^***^
RNT	SE	0.42	91.09^***^	0.62	8.58^***^
PSB	SE	0.37	46.94^***^	−0.01	−0.15
	RNT			−0.60	−8.50^***^

PSB, prosocial behavior; SE, social exclusion; RNT, relational need threat. ***p < 0.001.

The current results supported the viewpoint that social exclusion decreases prosocial behavior (Twenge et al., 2007). Furthermore, our findings suggested that social exclusion decreased prosocial behavior by threatening individuals’ levels of relational need, which was consistent with the findings of previous research (Williams, 2009). It is possible that social exclusion leads individuals to perceive their relationships with groups or individuals negatively due to compromised social relationships. Thus, individuals may not need to remedy their blocked needs by engaging in prosocial behaviors (Cuadrado et al., 2016; Moscardino et al., 2020).

#### 2.3.3 The moderated mediation effect of regulatory focus

After establishing the indirect effect of relational need threat on the relationship between social exclusion and prosocial behavior, we employed the moderated mediation model (Model 14) in the SPSS macro developed by Hayes (2013) to investigate whether this mediating effect was moderated by promotion orientation and prevention orientation.

Specifically, the results demonstrated that the main effects demonstrated that promotion orientation had a significant positive predictive effect on prosocial behavior (*β* = 0.14, *t* = 3.25, *p* < 0.01, 95% CI [0.06, 0.23]), whereas the effect of prevention on prosocial behavior was not significant (*β* = 0.03, *t* = 0.90, *p* > 0.05). Notably, the interaction between promotion orientation and relational need threat significantly predicted prosocial behavior (*β* = 0.15, *t* = 4.74, *p* < 0.001, 95%CI [0.09, 0.21]). Moreover, the interaction between prevention orientation and relational need threat negatively predicted prosocial behavior at a statistically significant level (*β* = −0.13, *t* = −4.20, *p* < 0.001, 95%CI [−0.20, −0.07]). These findings suggest that both promotion and prevention orientations moderate the predictive impact of relational need threat on prosocial behavior (see Table 3).

**Table 3:** Testing the moderated mediation effect of promotion orientation and prevention orientation.

Regression equation		Fitting index		Coefficient significance	
Outcome variable	Predictor variable	*R^2^*	*F*	*β*	*t*
RNT	SE	0.42	91.10^***^	0.62	8.58^***^
PSB	SE	0.44	49.39^***^	0.10	1.65
	RNT			−0.62	−9.62^***^
	Promotion			0.14	3.25^**^
	RNT × Promotion			0.15	4.74^***^
PSB	SE	0.40	38.60^***^	−0.02	−0.33
	RNT			−0.54	−9.08^***^
	Prevention			0.03	0.90
	RNT × Promotion			−0.13	−4.20^***^

PSB, prosocial behavior; SE, social exclusion; RNT, relational need threat. **p < 0.01; ***p < 0.001.

**Figure 2:**
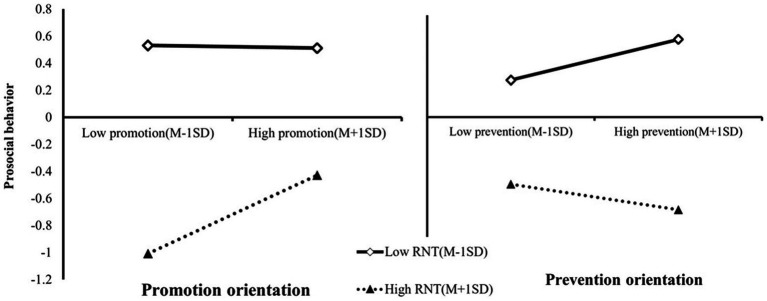
Plot of the relationship between regulatory focus and prosocial behavior at two levels of RNT.

Given that the interactions are statistically significant, we have conducted further simple slope analysis specifically targeting the two different regulatory orientations (Figure 2).

For the promotion orientation condition, we found that the predictive effect of low relational need threat (M-1SD) on prosocial behavior was no longer significant (*simple slope* = −0.01, *t* = −0.158, *p* > 0.05). Conversely, high relational need threat (M + 1SD) had a significant positive predictive effect on prosocial behavior (*simple slope* = 0.29, *t* = 7.25, *p* < 0.001). This suggested that promotion orientation could bolster individuals facing intense relational need threats, resulting in more prosocial behaviors.

In contrast, for the prevention orientation condition, high relational need threat (M + 1SD) failed to significantly predict prosocial behavior (*simple slope* = −0.10, *t* = −1.62, *p* > 0.05). However, a low relational need threat (M-1SD) had a significant positive predictive effect on prosocial behavior (*simple slope* = 0.16, *t* = 5.06, *p* < 0.001). This result indicated that, within the context of social exclusion, a prevention orientation was conducive to fostering prosocial behaviors among individuals facing minor relational need threats. Unfortunately, once the relational need threats became severe, the positive effect was no longer evident.

### 2.4 Discussion

In conclusion, in Study 1, we investigated the association between social exclusion and prosocial behavior via a questionnaire. The findings confirmed that the relationship between social exclusion and prosocial behavior is fully mediated by relational need threat, in line with previous findings (Yao and Enright, 2021). Our findings contribute to elucidating the differential consequences of social exclusion on the emergence of prosocial behavior.

Furthermore, our study revealed that promotion and prevention orientations moderated the impact of relational need threat on prosocial behavior. Individuals with a high promotion orientation exhibit increased engagement in prosocial behavior when faced with high relational need threats, while those with a strong prevention orientation display more prosocial behavior when relational need threats are low. This suggests that regulatory focus significantly influences individuals’ cognition, affective, and behavior. Individuals with a promotion orientation focus on potential gains and anticipate engaging in prosocial behavior to meet their threatened relational needs. Conversely, individuals with a high prevention focus aim to avoid losses and are less likely to engage in prosocial behavior unless it posed no ‘loss’ to their own needs (Higgins et al., 2001).

## 3 Study 2

Despite good reliability in self-report surveys, common method bias might have exaggerated the relationships between variables. To address this, we employed an experiment to further test the hypotheses in Study 2. In addition, considering that regulatory focus can also be triggered by situational factors and temporarily present, in Study 2, we adopted laboratory experiments to manipulate regulatory focus (promotion and prevention) to further validate Hypotheses 1 and 2.

### 3.1 Method

#### 3.1.1 Participants

We recruited a separate independent sample of 100 participants (58 female, aged 20.20 ± 2.20 years), which was entirely distinct from the one utilized in Study 1. Upon arrival at the laboratory, participants were instructed to sign informed consent forms. Subsequently, they were randomly assigned to one of four groups, following a 2 (exclusion manipulation: exclusion vs. inclusion) × 2 (regulatory focus: promotion orientation vs. prevention orientation) design. The experiment was conducted in a quiet room, and the gender distribution was balanced using a control program. Upon completion of the experiment, the participants were provided with an explanation of the actual purposes of the study and received a $5 equivalent compensation.

We estimated the sample size by G*Power (version 3.1; Faul et al., 2009), targeting large effect sizes (Cohen’s *f* = 0.40, *α* = 0.05, 1 – *β* = 0.95) for fix effects, special, main effects and interactions (ANOVA; Faul et al., 2009). The results showed that 84 participants were sufficient to detect a reliable effect. To ensure the effectiveness of the experiment, 100 participants were ultimately enrolled in this study, satisfying the requirement for the anticipated effect size.

#### 3.1.2 Measures

##### 3.1.2.1 Relational need threat

We employed the Fundamental Need Threat Scale developed by Wirth et al. (2010). The scale consisted of 10 items, with responses rated on a 5-point Likert scale, ranging from “1 = not true” to “5 = certainly true.” The Cronbach’s alpha coefficient for internal consistency is 0.91. Additionally, the variables of relational need threat were categorized into high and low groups using mean ± standard deviation.

##### 3.1.2.2 Prosocial behavior

We used the Prosocial Behavioral Tendencies Measure paradigm developed by Twenge et al. (2007) to measure prosocial behaviors. After completing the questionnaire, the participants were informed, “You can either leave now and receive credit for one hour of participation in the experiment, or you can assist me and other experimenters by completing additional tasks. Each additional task took approximately 5 min, and you could perform one, two, or three additional tasks. Your decision will not affect the amount of credit you receive. What you do is up to you.” The number of extra tasks participants volunteered for (ranging from zero to six) served as the indicator of prosocial behavior.

##### 3.1.2.3 Regulatory focus priming task

Dual priming tasks, including a self-guided task and a “mouse and maze” task, were used to prime participants in the regulatory orientation test. The self-guided task required participants to write down their previous and current expectations and ambitions to induce a promotion orientation and to write down their duties and obligations to induce a prevention orientation. The “mouse and maze” task, a pencil-and-paper maze task, was designed to activate promotion and prevention orientations by requiring participants to help a mouse either escape from a maze to eat cheese or avoid an eagle, respectively (Friedman and Forster, 2001).

##### 3.1.2.4 Social exclusion

We have taken and adapted from Wirth et al. (2010) as a manipulation check for social exclusion. 6 items are made on a 5-point Likert scale.

#### 3.1.3 Procedure

First, we manipulated social exclusion and inclusion using the “Get acquainted” paradigm. After providing informed consents, the participants were randomly grouped within the laboratory, each group comprising four people who were previously unacquainted. These groups then engaged in a 15-min discussion centered on a predetermined topic, allowing quick familiarization among members. After the discussion, the participants were assigned to positions where they could not see each other. The participants were subsequently asked to select two partners from their group for harmonious collaboration in upcoming tasks. Following this, the experimenter pretended to tally the number of nominations each participant received and gave each participant false feedback.

Participants in the exclusion group were informed that they had not been selected by anyone and were directed to complete a separate task instead of cooperating with other members for the next step. In contrast, participants in the inclusion group were informed that they had been chosen by all members, enabling them to proceed to the next experiment. Subsequently, they were instructed to complete a series of measurements. During this phase, we collected data on social exclusion and relational need threat. Simultaneously, we recorded the pre-test data of regulatory focus and prosocial behavior as the baseline level.

Then, participants in each group of social exclusion and inclusion were randomly assigned to two gender-matched subgroups, receiving either a regulatory focus prime or no prime treatment through the “mouse and maze” task. Specifically, two different sets of pictures and instructions were presented. One set was designed to prime a promotion focus by instructing participants to help the mouse escape the maze to reach the cheese, while the other set was aimed at priming a prevention focus by asking participants to aid the mouse in evading a hawk. After the task, we measured the participants’ regulatory focus and collected posttest data on prosocial behavior.

Finally, we debriefed the participants to mitigate any potential negative impacts stemming from the social exclusion experiment. Participants then were compensated for participating in the study.

### 3.2 Results

#### 3.2.1 Manipulation check

##### 3.2.1.1 Social exclusion

A t-test was also conducted to evaluate the effectiveness of the social exclusion manipulation. As hypothesized, excluded participants (*M* = 15.96, *SD* = 4.13) reported more negative feelings than did included participants (*M* = 27.64, *SD* = 3.41), *t_(98)_* = −15.48, *p* < 0.001, *d* = 3.10. Therefore, the manipulation of social exclusion in the experiment was effective.

##### 3.2.1.2 Regulatory focus

A t-test was also conducted to assess the effectiveness of the regulatory focus manipulation. As expected, participants primed with a promotion orientation had significantly greater scores (*M* = 15.15, *SD* = 3.28) on the three items than did those primed with a prevention orientation (*M* = 8.81, *SD* = 3.63), *t*_(98)_ = −9.17, *p* < 0.05, *d =* 1.84. Thus, the priming of regulatory focus was valid.

#### 3.2.2 Impact of social exclusion on relational need threat

A t-test was conducted, employing social exclusion (exclusion, inclusion) as the independent variable and relational need threat as the dependent variable. The results revealed that excluded participants (*M* = 24.68, *SD* = 5.81) had significantly greater relational need threat than did the included participants (*M* = 19.53, *SD* = 4.95), *t*_(98)_ = 4.80, *p* < 0.001, Cohen’s *d* = 0.96. This indicated that participants in the excluded condition experienced a more severe relational need threat.

#### 3.2.3 Impact of social exclusion and regulatory focus on prosocial behavior

**Figure 3:**
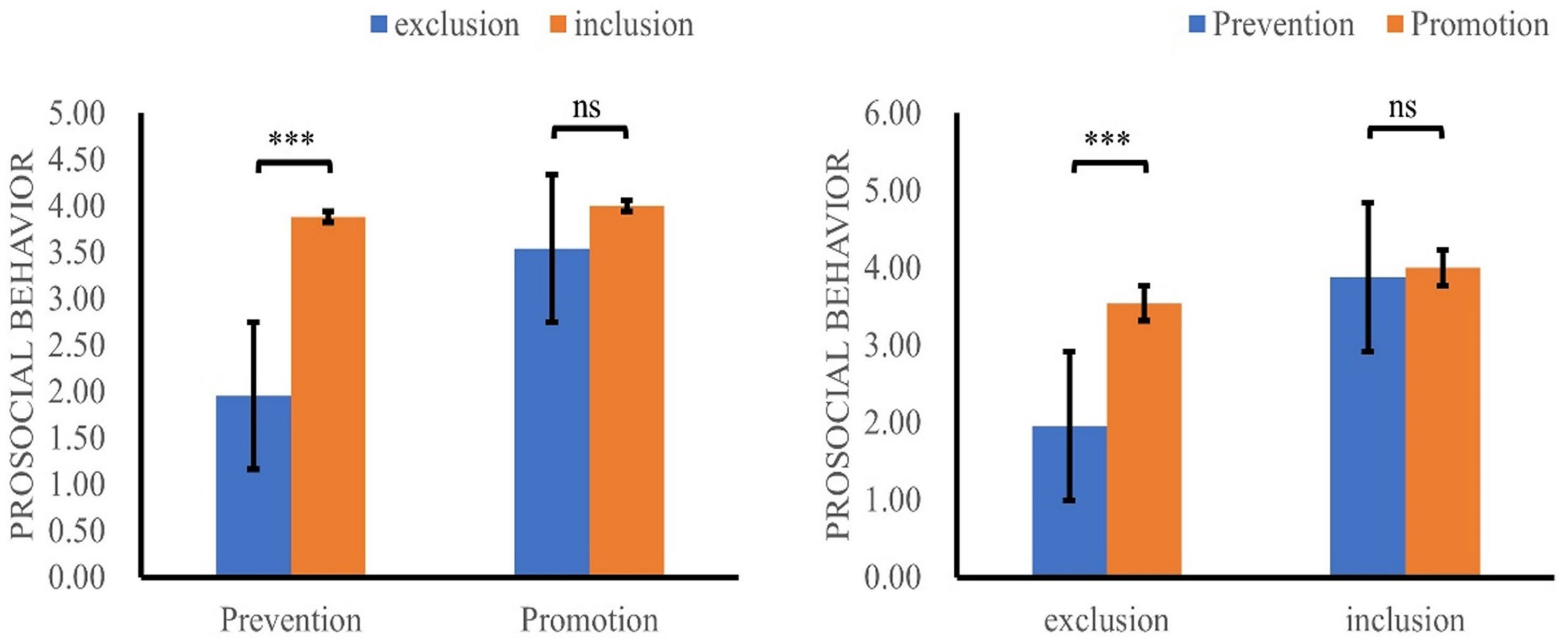
The interaction between social exclusion and regulatory focus on prosocial behavior.

A preliminary 2 (context: exclusion vs. inclusion) × 2 (regulatory focus: promotion vs. prevention) between-subject analysis of variance (ANOVA) was conducted. The results showed that the main effects of social exclusion [*F*(*_1, 96_*) = 16.00, *p* < 0.001, *η_p_^2^* = 0.14] and regulatory focus [*F*_(1, 96)_ = 8.20, *p* < 0.01, *η_p_^2^* = 0.08] were both significant. Importantly, the interaction between social exclusion and regulatory focus was also significant [*F*(_1, 96_) = 6.06, *p* < 0.05, *η_p_^2^* = 0.06] (Figure 3). Further simple effects analysis revealed that participants with a promotion orientation (*M* = 3.54, *SD* = 0.30) exhibited more prosocial behavior than did those with a prevention orientation (*M* = 1.96, *SD* = 0.31) in the excluded condition [*F*_(1, 96)_ = 13.39, *p* < 0.001, *η_p_^2^* = 0.12]. However, there was no significant difference between participants who received a promotion (*M* = 4.00, *SD* = 0.28) or prevention (*M* = 3.88, *SD* = 0.30) orientation in the included condition, *F* < 1.

Additionally, participants in the exclusion condition (*M* = 1.96, *SD* = 0.31) exhibited less prosocial behavior than did those in the inclusion condition (*M* = 3.88, *SD* = 0.30) when they were primed with a prevention orientation [*F*_(1, 96)_ = 20.11, *p*<0.001, *η_p_^2^* = 0.17]. However, when participants were primed for promotion, their prosocial behavior was not significantly different from that of participants in the included condition [*F*_(1, 96)_ = 1.23, *p* > 0.05, *η_p_^2^* = 0.01]. This suggested that promotion orientation was more beneficial for prosocial behavior in individuals experiencing social exclusion, consistent with the findings of Study 1.

#### 3.2.4 Testing for the moderated mediation model

We used the same moderated mediation model analysis as in Study 1. The results showed that the R^2^ for the entire model was 0.37, and the mediating effect of relational need threat was significant (*a* = 0.44, *t* = 4.70, *p* < 0.001; *b* = −0.39, *t* = −4.61, *p* < 0.001). The relational need threat fully mediated the relationship between social exclusion and prosocial behavior, *a* × *b* = −0.17, 95% CI [−0.31, −0.08] (see Table 4).

**Table 4:** Testing for the moderated mediation model.

Regression equation		Fitting index		Coefficient significance	
Outcome variable	Predictor variable	*R^2^*	*F*	*β*	*t*
RNT	SE	0.21	12.35^***^	0.42	4.54^***^
PSB	SE	0.37	17.32^***^	−0.15	−1.51
	RNT			−0.39	−4.41^***^
	RF			0.47	2.82^**^
	RNT × RF			0.49	3.25^**^

PSB, prosocial behavior; SE, social exclusion; RNT, relational need threat; RF, regulatory focus. **p < 0.01; ***p < 0.001.

The interaction between relational need threat and regulatory focus significantly predicted prosocial behavior (RNT × RF: *β* = 0.25, *t* = 3.25, *p* < 0.01), implying that regulatory focus moderates the effect of relational need threat on prosocial behavior.

**Figure 4:**
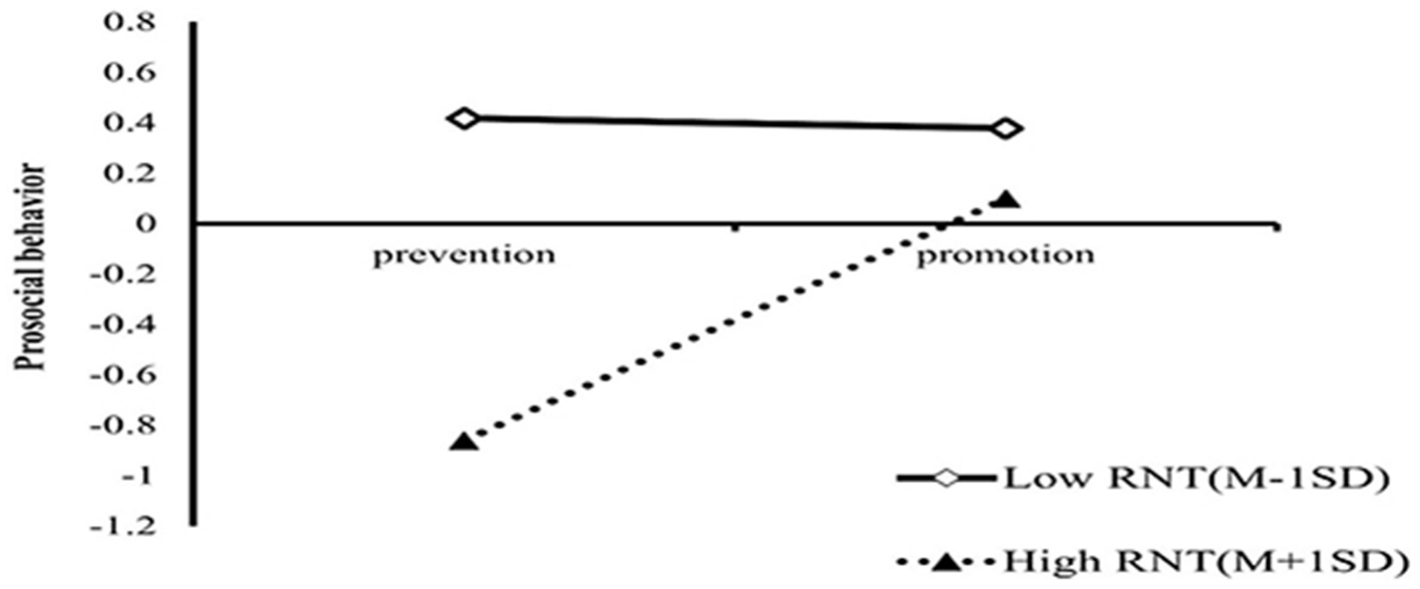
Interaction effect of relational need threat and two type orientations on prosocial behavior.

Further simple slope analysis results revealed that (Figure 4), the changes in prosocial behavior among different levels of relational need threat were not the equivalent in the two regulatory orientations. For participants with low levels of relational need threat, there was no significant change in prosocial behavior between the two regulatory orientations (*simple slope* = −0.01, *t* = −1.10, *p* = 0.92). In contrast, when the relational need threat was high, a significant change in prosocial behavior was observed among participants with different regulatory orientations (*simple slope* = 0.47, *t* = 3.92, *p* < 0.001). Specifically, this means that for high relational need threat participants, the promotion orientation condition will be more beneficial in enhancing their prosocial behavior. This indicates that individuals with promotion orientation are more inclined to engage in prosocial behavior after social exclusion, and they attempt to restore threatened relationship needs through positive social interactions. Conversely, individuals with prevention orientation tend to reduce social interactions to avoid the recurrence of social exclusion.

### 3.3 Discussion

Study 2 further explored the role of regulatory orientations in a laboratory setting, providing more objective, reliable, and ecologically valid data. We used a “Get acquainted” paradigm to manipulate social exclusion and a dual-task paradigm to initiate promotion and prevention orientations. Study 2 further supported the findings of Study 1. Excluded individuals exhibited lower prosocial behavior than included individuals did, and regulatory focus played a crucial moderating role in the relationship between social exclusion and prosocial behavior. Specifically, in the excluded condition, participants with a promotion orientation exhibited significantly greater prosocial behavior than those with a prevention orientation. This indicates that individuals with promotion focus are more willing to engage in prosocial behavior after social exclusion, attempting to restore threatened relational needs through positive social interactions.

Conversely, individuals with prevention focus tend to reduce social interactions to avoid further exclusion. Moreover, no significant difference was found in the prosocial behavior of those with promotion or prevention orientations in the included condition. These results are consistent with previous findings (Adams and Tyler, 2021).

Furthermore, Study 2 also validated the moderated mediation model. The mediating effect of relational need threat was significant, and the moderated effect of regulatory focus was significant in the second half of the path. More importantly, the results of the simple slope analysis showed that the prosocial behavior of subjects with high relational need threat significantly differed across different orientations of regulatory focus.

To be specific, the prosocial behavior level in the promoting orientation improved. This result is consistent with the findings of Study 1. In contrast, the prosocial behavior of participants with low relational need threat did not significantly differ between the two kinds of regulatory orientations. This finding did not replicate the result of Study 1, which suggested that a prevention focus was more beneficial in increasing prosocial behavior. This may be related to the fact that the level of prosocial behavior of participants with low relational need threat did not differ significantly between both regulatory orientations in Study 2.

## 4 General discussion

College students who experience social exclusion suffer serious impacts on their physical and mental health development, leading to academic and social disadvantages, as well as feelings of loneliness and depression (Arslan, 2018). Nevertheless, it was noteworthy that such exclusion can elicit diverse behavioral outcomes, encompassing both aggression and prosocial behavior (Twenge et al., 2007; Killian et al., 2023). The latter behavior is beneficial for enhancing individual adaptability (Bian and Wu, 2023). The present study, employing college students as participants, verified the underlying mechanisms of the impact of social exclusion on prosocial behavior based on the temporal need-threat model and the CAPS theory framework.

This research yielded several noteworthy findings. Study 1, employing questionnaires, revealed that social exclusion significantly and negatively predicted prosocial behavior, with relational need threat fully mediating the relationship. Our findings were consistent with previous studies indicating that social exclusion reduces individuals’ prosocial behavior (Yao and Enright, 2021) and provided explanatory insights into this phenomenon. Additionally, different types of regulatory orientations played varying moderating roles in this mediation model. Specifically, a promotion orientation promoted more prosocial behavior among participants with high relational need threat. Instead, a prevention orientation was beneficial for prosocial behavior only at lower levels of relational need threat. Building upon these findings, Study 2 conducted in the laboratory further corroborated the results of Study 1: In the exclusion condition, individuals with a promotion orientation exhibited more prosocial behavior than did those with a prevention orientation.

Our combination of questionnaire and laboratory methods provided robust sample and experimental evidence supporting the hypothesis that social exclusion reduces prosocial behavior. Moreover, our study provides fresh perspectives on the theoretical explanation of prosocial behavior. While previous research has identified situational variables (such as social exclusion), relational need threat, and personality (regulatory orientation) as predictors of prosocial behavior (Howard et al., 2020; Moscardino et al., 2020), the interaction between these variables has remained largely unexplored, leading to a fragmented understanding. Our study adopted an integrative approach, proposing the CAPS theory as a complementary explanatory framework alongside the temporal need-threat model. Specifically, we propose that the events individuals encounter interact with their complex cognitive-affective system to shape their behavior (Mischel and Shoda, 1995). Emotion, a key component of this cognitive-affective unit, can suppress some behaviors (e.g., prosocial behavior) or prompt them in specific situations (e.g., social exclusion), depending on individual expectations and beliefs (e.g., expectations of the behavior-outcome relationship). This perspective provides an alternative explanation for the prosocial behavior of individuals experiencing severe relational need threats and aspiring for social reconnection. Specifically, prosocial behavior may be induced by individuals’ expectations regarding the relationship between behavior and outcome.

Importantly, this perspective highlights the crucial role of personality in determining individual differences in behavior across various situations. This finding offers a credible explanation for the notion that a promotion orientation could more positively predict prosocial behavior than a prevention orientation, due to its stronger emphasis on progress and positive outcomes. Individuals with a promotion orientation are more inclined to engage with group members, experience positive emotions, and thus are more likely to exhibit prosocial behavior.

Furthermore, we acknowledge that previous literature has presented conflicting findings on whether prosocial behavior increases or decreases following social exclusion (Twenge et al., 2007; Howard et al., 2020). We believe that the regulatory focus variable can provide a new perspective to explain the contradictory result of the influence of social exclusion on prosocial behavior. According to CAPS, individual behaviors are guided by interactions between personality and other internal variables. Our results consistently demonstrated that individuals did increase prosocial behavior in response to social exclusion, which is related to different regulatory orientations of self. The findings of Study 2 indicated that reactions to exclusion can either enhance or diminish prosocial behavior, depending on the individual’s self-regulatory styles. To be specific, highly promotion-oriented individuals tend to self-regulate in response to social exclusion by focusing on personal gains (DeWall et al., 2011). When their relational needs were threatened, these individuals strived for harmony and satisfaction. They often adjusted their behavior to either compensate for or mend their relationship with others, which led to an increase in prosocial behavior (Williams, 2007; Bakhti et al., 2022). In summary, the promotion orientation of regulatory focus has a positive impact on prosocial behavior, and this effect is robust regardless of trait or situational condition.

Moreover, our questionnaire survey found that prevention-oriented individuals, who focused more on potential losses and threats, typically adjusted their behaviors to minimize self-harm facing high relational need threats. Therefore, to avoid more severe negative situations, these individuals were prone to adopting a “vigilance-avoidance” strategy and displaying reduced prosocial behavior to ensure safety (Bakhti et al., 2022). However, it is still more additional evidence, especially from the laboratory experiments, would provide beneficial support.

## 5 Implications and limitations

Our research aims to assist practitioners in identifying that can promote college students’ prosocial behavior and guide specific cultivational pathways for students encountering social exclusion. Given our study results, relational need threat and regulatory focus are two crucial factors that impacting the relationship between social exclusion and prosocial behavior. On one hand, researchers can attempt to improve students’ prosocial behavior by meeting their relational needs. Specifically, based on the temporal need-threat model, counseling teachers and parents can help individuals to enhance self-esteem and foster a sense of belonging, thus reducing the relational need threat caused by social exclusion. For students suffering from social exclusion, teachers can carry out targeted interventions, guiding them to seek social support from relationships with family, friends, etc., enhance individual sense of belonging, and aiding in the restoration of threatened relation needs, thereby reducing the negative behavioral responses induced by social exclusion.

On the other hand, our research findings demonstrate that self-regulation tendency can influence individuals’ behavioral responses after being excluded, providing guidance for students encountering social exclusion. In order to help students regain social connections, educators can focus on cultivating students’ self-regulation strategies, assisting excluded individuals in shifting their perspectives, solving problems in an open and optimistic manner, and adopting a positive attitude to cope with social exclusion. Furthermore, educators have the capability to improve students’ propensity toward promotion orientation by implementing group interventions and utilizing role models. They can encourage and reward prosocial behavior accordingly, thereby internalizing it as an aspect of their personal values. By executing these strategies, they can efficiently cultivate students toward fostering positive social behaviors, ultimately promoting societal harmony and advancement.

Some limitations of this study should be acknowledged. First, this study mainly examined the effects of the regulatory focus on their behavioral responses after encountering rejection. Previous studies have also found that individuals will transform from a promoting motivation to a preventing motivation after being ostracized (Park and Baumeister, 2015). Therefore, it remains worthwhile to further explore the specific mechanisms of individual pre-rejection and post-rejection regulatory focus on prosocial behavior. Second, in our study 2, the get acquainted paradigm was adopted in the experimental research, which was mainly related to rejection situations. Previous studies have divided social exclusion into neglected and rejected scenarios which may threaten different basic needs and consequently lead to differences in individual behavioral responses (Lee and Shrum, 2012). Therefore, we can continue to explore whether there are differences in the influence of regulatory orientation on prosocial behavior under different types of exclusion. Finally, this study used college students as participants, so the generalizability of the conclusions still needs verification and should be cautiously extended to other age groups.

## 6 Conclusion

1. After experiencing social exclusion, individuals can exhibit prosocial behavior, but the occurrence of such behavior depends on the threat level to their basic relational needs.
2. The promotion orientation of regulatory focus robustly moderates the relationship between relational need threat and prosocial behavior. The higher the level of promotion orientation, the more beneficial it is for prosocial behavior.

## Data availability statement

The raw data supporting the conclusions of this article will be made available by the authors, without undue reservation.

## Ethics statement

The studies involving humans were approved by the Ethics Committee of School of Psychology at Northeast Normal University. The studies were conducted in accordance with the local legislation and institutional requirements. Written informed consent for participation in this study was provided by the participants’ legal guardians/next of kin. Written informed consent was obtained from the individual(s) for the publication of any potentially identifiable images or data included in this article.

## Author contributions

CH: Funding acquisition, Supervision, Writing – original draft, Writing – review & editing. SL: Data curation, Writing – original draft. HS: Writing – review & editing, Software. ZL: Formal analysis, Project administration, Supervision, Writing – review & editing.
